# Isolation and selection of indigenous chicken-derived *Bacillus subtilis* strains as potential probiotic alternatives to antibiotics against Gram-negative enteropathogens

**DOI:** 10.5455/javar.2025.l871

**Published:** 2025-03-24

**Authors:** Hai Vu Phan, Hung Hoang Son Pham, Lai Huu Ngo, Na Thi Tran, Dung Thi Ho, Khuong Dinh Thuy Nguyen, Liem Ngoc Tran, Hoa Xuan Nguyen

**Affiliations:** 1 Department of Animal Husbandry and Veterinary Medicine, College of Agriculture and Forestry, Hue University, Hue City, Vietnam; 2 Region IV, Vietnam Department of Animal Health, Da Nang City, Vietnam

**Keywords:** Antibiotic resistance, *Bacillus subtilis*, *E. coli*, Probiotic, *Salmonella* spp.

## Abstract

**Objective:**

The increasing antibiotic resistance in poultry pathogens presents a significant public health risk, leading to the exploration of effective alternatives in broiler feed, particularly probiotics such as *Bacillus subtilis*. This study aimed to isolate *B. subtilis* strains from indigenous chicken feces that can inhibit *Escherichia coli* and *Salmonella typhimurium *strains, common causes of diarrhea in poultry.

**Materials and Methods:**

*Bacillus* strains were isolated from chicken feces and screened for antibacterial activity using an agar well diffusion assay. *Bacillus* strains were identified via 16S rRNA gene sequencing. Their probiotic potential was assessed through *in vitro* assays measuring extracellular enzyme production, adhesion properties, and resilience to acidic and bile salt conditions. Freeze-drying techniques were applied to evaluate strain viability and stability. *In vivo* studies determined the colonization ability of selected strains in the chicken intestine.

**Results:**

From 121 *B.* *subtilis* isolates, six *B. subtilis* strains demonstrated notable antibacterial activity against both *E. coli* and *S. typhimurium*. Five strains were confirmed as *B. subtilis* through sequencing. Based on their probiotic attributes, *B. subtilis* H1 and *B. subtilis* BSn5 were identified as the most promising candidates. Notably, *B. subtilis* BSn5 exhibited stable viability when freeze-dried, surviving for up to two months, and successfully colonized the chicken intestinal tract *in vivo*.

**Conclusion:**

These findings indicate that *B. subtilis* BSn5 may serve as a viable probiotic alternative to antibiotics in poultry, with regular supplementation necessary to sustain its benefits.

## Introduction

Currently, *Escherichia coli* and *Salmonella* spp. are prevalent Gram-negative bacteria that cause gastrointestinal diseases in poultry, leading to substantial economic losses for the poultry industry [1]. These pathogens, particularly, have zoonotic potential, posing a serious public health threat through foodborne transmission [2]. At present, there are no effective vaccines to protect chickens against these infections due to the diversity of bacterial serotypes and limited cross-protection [3]. Both *E. coli* and *Salmonella* spp. are listed by the WHO as pathogens for which antibiotic susceptibility testing is recommended before treatment [4]. The rising issue of antibiotic resistance, fueled by the overuse of antibiotics in livestock, has resulted in widespread resistance in *E. coli* [5] and *Salmonella* spp. [6], diminishing antibiotic efficacy, altering the gut microbiota, and negatively impacting health. Consequently, the search for antibiotic alternatives, such as probiotics, has become imperative.

Probiotics comprise live microorganisms and their metabolites, which, when administered orally, confer health benefits to the host. In addition to producing antimicrobial compounds such as organic acids and bacteriocins, probiotics can compete with pathogenic bacteria by adhering to the intestinal epithelium, thereby preventing pathogen colonization, disrupting cell-to-cell communication, and inhibiting biofilm formation and virulence [7]. Unlike antibiotics, probiotics do not induce resistance, leave no residues, and are environmentally friendly [8]. Among various probiotics, bacteria from the *Bacillus* genus are widely used in both humans and animals due to their antimicrobial compound production and spore-forming ability, which provides a dual advantage for survival in diverse environments [9].

Notably, *Bacillus subtilis* strains are garnering interest as feed additives due to their beneficial impact on animal health, as these bacteria can form spores that withstand harsh conditions, such as high pH, acidic environments, and elevated temperatures [10]. This resilience probably allows *B. subtilis* to survive the extreme conditions of the gastrointestinal tract, enhancing its stability during production, storage, and feed formulation while also extending shelf life and increasing gastrointestinal stability in animals. In commercial probiotic products, the efficacy of *B. subtilis* varies by strain, and the specific characteristics of these strains are often not fully documented. This lack of detail can lead to mislabeling of products and may result in the inclusion of strains that contain harmful factors, such as toxins, posing risks to both animal and public health.

Moreover, numerous researchers emphasize that probiotics isolated from the host organism offer a better chance of survival and efficacy compared to those derived from other sources, as they are more likely to overcome the challenges associated with introducing foreign bacteria [11]. This study is the first to focus on the isolation and selection of potential *B. subtilis* from free-ranging chicken feces to combat Gram-negative pathogens (*E. coli* and *Salmonella* spp.) responsible for diarrhea in broilers, based on their antimicrobial capabilities and probiotic properties.

## Materials and Methods

### Ethical approval

In this study, all procedures related to the care, housing, and slaughtering of experimental chickens were conducted following the standards and approvals of the Animal Ethics Advisory Committee, Hue University, Vietnam (Approval No: HUVNO39).

#### Isolation of Bacillus spp.

*Bacillus* spp. was isolated from the feces of healthy, semi-free-range indigenous broiler chickens (Ga Kien) that had not been supplemented with probiotics. The chickens were provided with a healthy and enjoyable diet that meets all their nutritional needs while allowing them to express their natural foraging behaviors. The isolated strains were screened using serial dilution and heat shock treatment in a water bath (Unitronic^®^ 300) at 80°C for 10 min, as described by Cazorla et al. [12], to eliminate vegetative cells, retaining only spore-forming strains for *Bacillus* isolation. Subsequently, 0.1 ml of each sample was streaked onto nutrient agar plates (Merck, Germany) and incubated for 24 h at 37°C. Single colonies, selected based on morphological differences, were transferred to fresh plates until consistent monocultures were obtained after three rounds of subculturing. Morphologically distinct colonies were purified, examined for Gram staining, and subjected to biochemical tests, including lactose, glucose, mannitol, and xylose fermentation; starch hydrolysis; gas production; motility; indole; urease; catalase; H_₂_S production; and Voges–Proskauer, as outlined in the Manual of Systematic Bacteriology. Pure colonies were then cultured in LB broth, with the pure cultures preserved in Eppendorf tubes supplemented with 40% glycerol and stored at –80°C.

### Species identification of Bacillus using API 50 CHB Kit

The API 50 CHB kit (bioMérieux, France), consisting of 50 biochemical tests, was used to identify *Bacillus* strains at the species level. Following incubation, the bacteria were introduced into the kit wells. After 24–48 h of incubation, results were interpreted based on color changes: positive (red to yellow) or negative (no color change). The outcomes of the 50 reactions were input to the API web software to identify the species and determine the similarity percentage.

### Evaluation of antibacterial activity against chicken gastrointestinal pathogens

Pathogenic bacteria: *Escherichia coli* FG31-1 and *Salmonella typhimurium* FC13827 (GenBank IDs: CP142680.1 and MN704402.1), carrying the virulence genes *invA* and *stn*, were isolated from the diarrhea feces of chickens suspected of *E. coli* or *Salmonella* spp. infections. These isolates were first identified based on their phenotypic and biochemical characteristics, followed by confirmation through 16S rRNA gene sequencing (Table 2). These strains were maintained at the Microbiology Laboratory, Faculty of Animal Science and Veterinary Medicine, Hue University of Agriculture and Forestry. The antibacterial activity of selected *Bacillus* strains against these pathogens was assessed using the agar well diffusion method. Muller Hinton Agar (MHA, Thermo Fisher Scientific) plates with 4 mm height and 100 µl of the bacterial suspension were overlaid with 0.5 OD 630 pathogenic bacterial suspension. After allowing the suspension to settle for 15–20 min, six wells (with a diameter of 6 mm and spaced 30 mm apart) were made on the MHA plates by marking positions along a straight line and creating the wells at those marked points. Each well received 100 μl of overnight LB broth culture of the selected *Bacillus* strains (adjusted to 0.5 OD 630). The plates were allowed to diffuse for one hour at 4°C and then incubated at 37°C for 24 h. Evaluation formula: Diameter (D, mm) = Diameter of inhibition zone (DIZ). DIZ of ≥ 10 mm was considered indicative of antibacterial activity [13].

### Identification of suspected B. subtilis strains via gene sequencing

Suspected *B. subtilis* strains were identified through 16S rRNA gene sequencing. Genomic DNA from a single colony of each isolated strain was extracted using the Bacterial DNA Kit TM (Zymo Research, Cat. No. D6005, USA) targeting a 1,500 bp gene fragment. Amplification of the 16S rRNA gene was performed using universal primers with the sequences 27F (5’-AGA GTT TGA TCM TGG CTC AG-3’) and 1492R (5’-TAC GGY TAC CTT GTT ACG ACT T-3’) [14]. The thermal cycling conditions were 1 cycle at 94°C for 5 min, followed by 35 cycles at 94°C for 1 min, 55°C for 1 min, 72°C for 1 min, and a final extension at 72°C°C for 15 min. PCR products were purified using the ZR-96 DNA Sequencing Clean-up Kit (Zymo Research, USA) according to the manufacturer’s instructions and sequenced using the CLC 7 sequencing system (QIAGEN, Germany). The PCR products were stained with SYBR Green for visualization under ultraviolet light and electrophoresed on an agarose gel, with a DNA ladder included for size estimation of the PCR bands. After Sanger sequencing and BLAST (Thermo Fisher Scientific) analysis, the data were compared with the GenBank database of the NCBI for *Bacillus* species identification.

The phylogenetic tree of *B. subtilis* strains was constructed using Geneious Prime software (Biomatters, New Zealand), using the neighbor-joining method. The branch lengths correspond to the ladder with a mutation rate of 0.06 mutations/site.

### Probiotic properties analysis of B. subtilis

Amylase activity was measured qualitatively in starch agar and quantitatively by the DNS (dinitrosalicylic acid) method as described by Sharif et al. [15]. Amylase hydrolyzes in starch agar, which then reacts with DNS to form a colored complex, measured at 540 nm. Protease activity was quantified as directed by Zhang et al. [16], using the casein plate method with absorbance read at 660 nm. Lipase activity was determined according to Bharathi and Rajalakshmi [17], using triglycerides as the substrate. Lipase catalyzes the hydrolysis of triglycerides to release glycerol and free fatty acids, which were quantified by titration or colorimetric methods.

The hydrophobicity assay, based on Krausova et al. [18] with minor adjustments, assessed microbial adhesion to xylene. *Bacillus subtilis* cultures were grown in LB medium, centrifuged, washed with Ringer’s solution, and resuspended to ~0.08 OD at 600 nm. Xylene was added and vortexed, and the aqueous phase was evaluated at 600 nm after separation. The percentage hydrophobicity was calculated as follows: [(OD0—OD) / OD0] × 100, where OD0 and OD represent the optical densities before and after mixing with xylene.

Auto-aggregation and co-aggregation assays were conducted as described by Mallappa et al. [19]. *Bacillus subtilis* strains were centrifuged at 8,500 rpm for 10 min and then resuspended in phosphate-buffered saline and incubated at 37°C for 4 h. A 0.2 ml sample of the suspension was taken, and OD at 600 nm was measured before and after incubation. Auto-aggregation was measured using the formula 1—[At / Ao] × 100, where Ao and At are the initial and final optical densities. For co-aggregation, *B. subtilis* was prepared similarly. An *E. coli* suspension in BHI medium was standardized to approximately 1 × 10_8_ CFU/ml. *Bacillus* suspension was mixed with *E. coli* suspension (1 ml/1 ml) and shaken for 10 sec and then allowed to settle. A control containing only 2 ml of the bacterial suspension was prepared. The absorbance at 600 nm was measured after 5 h of incubation at 37°C. Co-aggregation was measured using the formula: (OD600 (x) + OD600 (y)—OD600 (x + y)) / (OD600 (x) + OD600 (y)) × 100.

Acid and bile tolerance of *B. subtilis* was assessed following the method by Mallappa et al. [19]. The *B. subtilis* strains were cultured overnight and suspended in 10 ml LB with pH adjusted to 2 and in 50 ml LB containing 0.3% bile salts (Himedia, India). The inoculum size was standardized to 0.5 McFarland (~1.5 × 10_8_ CFU/ml). The tubes were incubated at 37°C for 3 h, with 300 μl samples taken at 0, 1, 2, and 3 h for growth dynamics measurements using a spectrophotometer at 600 nm. Concurrently, 100 µl samples were taken to determine viable cell counts by the standard plate count method.

### Evaluation of viability of selected B. subtilis strains during storage

Freeze-drying of bacterial strains cultured on skim milk was conducted in a medium enriched with maltodextrin and trehalose. Twenty-four-hour cultures of each isolated strain (20 ml each) were transferred to sterile, disposable polypropylene containers (60 ml capacity). The samples were initially stored at –20°C for 72 h, followed by freeze-drying under vacuum conditions at a constant pressure of 63 Pa for 96 h, with a shelf temperature of 30°C on a lyophilized (Mactech MST50GD, Meta Company, Vietnam). The freeze-dried samples of the five bacterial strains were then ground and thoroughly mixed in equal proportions to produce a prototype feed additive.

The stability of the five freeze-dried strains and the feed additive was determined by comparing bacterial counts on plates immediately after freeze-drying and after 60 days of storage at 4°C. The samples were placed in sterile, sealed containers to evaluate their storage stability. The number of viable cells or spores was measured on agar plates both before and after freeze-drying and throughout two months of storage. The samples of each preparation form were rehydrated, serially diluted, and plated. Survival rates were evaluated based on Lo Curto et al. [20] with some modifications by exposing the probiotic powder to an HCl solution (pH 1.5, at 37°C for 2 h) and subsequently to a 0.05 M phosphate buffer (pH 7.4) at 37°C. All preparations were stored over silica gel in closed glass containers (desiccators) at 48°C for 2 months.

### Assessment of bacterial viability in the chicken gut

A total of 36 one-day-old 3FViet chicks were divided into two groups, each containing three cages (6 chicks per cage). The experimental group received 1 ml of preparation containing 10^9^ CFU/ml *B. subtilis *BSn5, while the control group was given orally 1 ml of distilled water by syringe. The chicks were housed in iron cages (0.9 × 0.5 × 0.5 m) under continuous light and maintained at 35°C throughout the experiment (1–3 days of age). Before the experiment, the cage system and floors were sterilized using heat (gas torch) and disinfectant (Povidone 10%).

The chicks were fed a diet of locally sourced ingredients such as rice bran, cornmeal, peanut meal, and soybean meal, meeting the standards set by the Ministry of Agriculture and Rural Development, Vietnam (10 TCN 661-2005). Before use, feed and water were sterilized by UV light (300 µW-s/cm², 30 min) and provided ad libitum to the chicks. At 24, 48, and 72 h, three chicks from each group (one per cage) were randomly selected and sacrificed, and samples were collected from the ileum, cecum, and colon. These samples were washed and heat-treated at 80°C for 20 min to eliminate vegetative cells and other bacteria. Finally, the counts of *B. subtilis* spores were determined at the specified time points. Results were expressed as the average number of spores per gram in the ileum, cecum, and colon.

### Statistical analysis

All assays were performed in triplicate, and data were expressed as mean ± standard deviation or percentage. Bacterial counts were converted to log_10_ CFU/ml. Statistical analysis was conducted using IBM SPSS software (version 22), with significance assessed by one-way ANOVA followed by Tukey’s post hoc test, and results were statistically significant at *α* = 0.05.

## Results and Discussion

### Isolation and identification of Bacillus spp.

#### Isolation of *Bacillus* spp.

A total of 121 bacterial isolates with morphological characteristics suggestive of *Bacillus *strains were obtained with the following characteristics: Gram-positive, opaque white, dry colonies with irregular edges, cell size greater than 3 µm, and the presence of centrally located endospores that did not distort the cell shape.

#### Biochemical characterization

Among the 121 isolates, 100% were catalase-positive, 58.7% (71/121) were VP-positive, 73.2% (52/71) were amylase-positive, 52.5% (40/52) were able to grow at 50°C, and 52.5% (21/40) were cellulase-positive. These 21 isolates were selected for further analysis.

### Identification using the API CH50B kit

**Figure 1.**
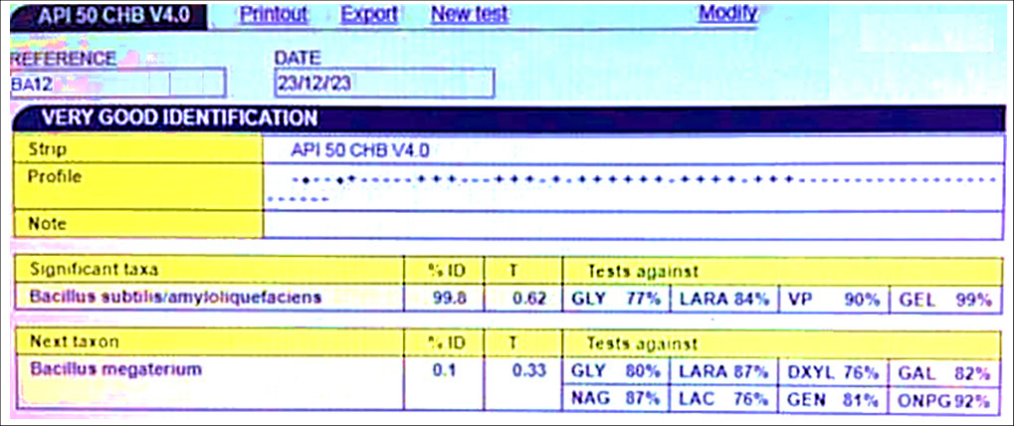
Reading the identification results using the API software.

Following biochemical characterization, 21 isolates exhibited characteristics consistent with *B. subtilis*. To confirm their taxonomic identity, these isolates were further analyzed using the API CH50B kit (Table 1, Fig. 1). The results revealed that 16 isolates were identified as *B. subtilis/B.* *amyloliquefaciens*, with a high degree of similarity (> 90%).

### Selection of B. subtilis strains based on their antibacterial properties on chickens

Grethel Milián et al. [20] reported that *Bacillus* strains C-31, C-34, and E-44 produced antimicrobial substances that completely inhibited the growth of indicator strains such as *Aerobacter*, *Staphylococcus*, *Klebsiella*, *Proteus*, *Listeria innocua*, *L. monocytogenes*, *S. aureus* 29737, *Klebsiella* 130300, *S. cholermidis* 12228, and *P. vulgaris* 13315.

The observed antibacterial activity can be attributed to the production of bacteriocins, antimicrobial peptides that suppress the growth of harmful bacteria by disrupting cell membrane integrity or interfering with protein or DNA synthesis [9]. Additionally, some *Bacillus* strains produce antibiotics such as polymyxin and gramicidin, which exhibit broad-spectrum antibacterial activity, including against *E. coli* and *Salmonella* spp. [22]. This antibacterial activity, coupled with competitive exclusion mechanisms, whereby probiotic strains compete with pathogens for nutrients and attachment sites, effectively prevents pathogen colonization in the gut.

**Figure 2.**
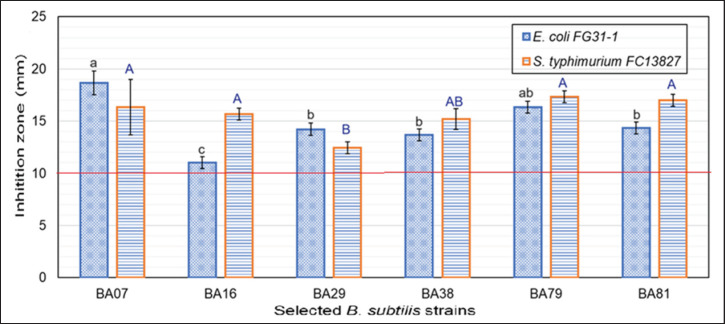
Antibacterial activity of selected *B. subtilis* strains. Values with different letters (a-c for *E. coli* or A, B for *S. typhimurium*) indicate statistically significant differences (*p *< 0.05).

The assessment of antibacterial activity is a crucial step in identifying potential probiotic candidates for antibiotic replacement. The antagonistic activity of 21 *B. subtilis* isolates against two Gram-negative enteropathogens were evaluated. As shown in Figure 2, six *B. subtilis *strains (28.57%) exhibited inhibitory activity against both pathogens, with DIZ ≥ 10 mm. Eight strains (38.09%) inhibited only one of the two pathogens, while seven strains (33.33%) did not exhibit inhibitory activity against either pathogen (data was not shown). Among the six strains with inhibitory activity against both pathogens, strain BA07 displayed the most potent activity, with significantly larger DIZ (16.4–18.2 mm) compared to the other strains (11.3–17.4 mm) (*p* < 0.05). Strains BA29, BA38, BA79, and BA81 exhibited relatively similar DIZ, ranging from 12.6 to 17.4 mm. *S. typhimurium* FC13827 appeared to be more susceptible to the B. subtilis strains than *E. coli* FG31-1, as evidenced by larger DIZ for 4 out of 6 *B. subtilis* strains.

**Table 1.** Identification of 21 bacterial isolates using the API CH50B kit.

Number of strains	Similarity ratio (%)	Isolate symbol	Species
2	99.9	BA07, BA91	*B. subtilis/B. amyloliquefaciens*
1	99.8	BA102	*B. subtilis/B. amyloliquefaciens*
2	99.3	BA12, BA56	*B. subtilis/B. amyloliquefaciens*
1	99.3	BA15	*B. subtilis/B. amyloliquefaciens*
2	98.7	BA38, BA92	*B. subtilis/B*. *amyloliquefaciens*
1	98.0	BA16	*B. subtilis/B. amyloliquefaciens*
1	98.0	BA81	*B. subtilis/B*. *amyloliquefaciens*
1	99.5	BA59	*B. licheniformis*
2	98.5	BA074, BA69	*B. licheniformis*
2	90.6	BA82, BA112	*B. licheniformis*

### Tentative identification of *B. subtilis* by gene sequencing

Agarose gel electrophoresis revealed that all selected strains yielded a 1,500 bp amplicon, consistent with the expected size of the 16S rRNA gene. The obtained 16S rRNA gene sequences were deposited in GenBank, and a phylogenetic tree was constructed based on these sequences.

**Figure 3.**
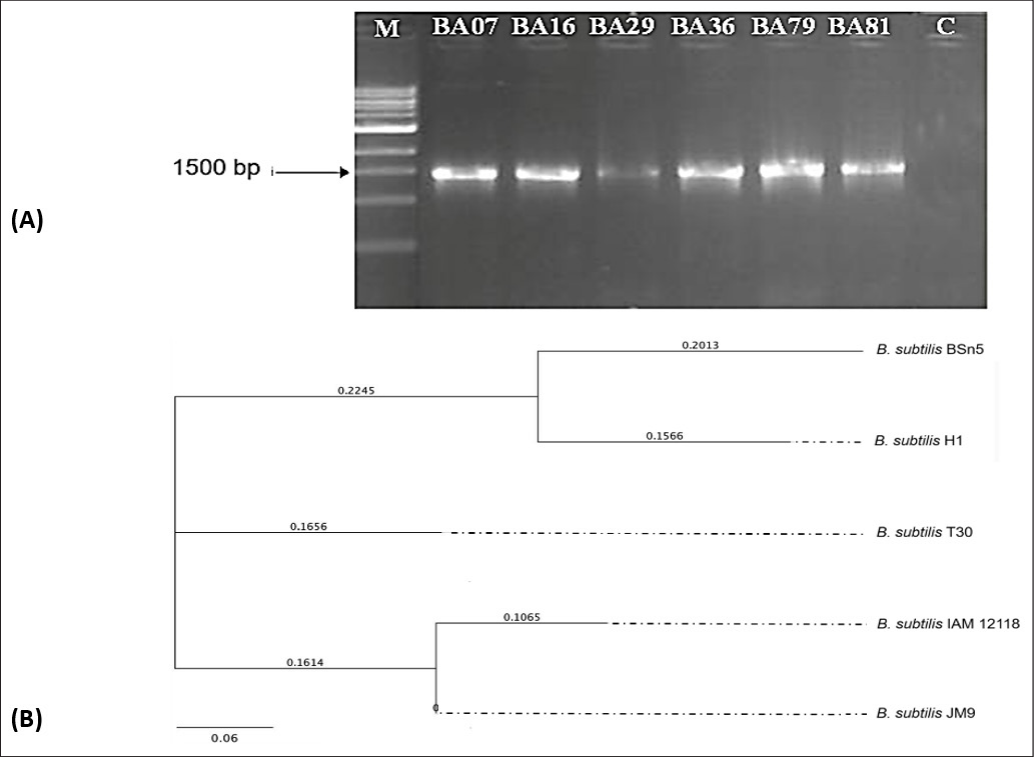
A: PCR products of *B. subtilis* strains used in this study (M: 100 bp DNA ladder; C: negative control). B: Neighbor-Joining phylogenetic tree of selected *B. subtilis* strains with an average nucleotide change rate of 0.06 mutations/site.

The six *Bacillus* strains, designated as BA7, BA16, BA29, BA36, BA79, and BA81, were identified as *B. subtilis* strain H1, *B. subtilis* strain IAM 12118, *B. subtilis* strain BSn5, *B. amyloliquefaciens* strain G341, *B. subtilis* ssp. strain JM9, and *B. subtilis* strain T30, respectively, through 16S rRNA gene sequencing (Table 2, Fig. 3). These sequences were compared against the NCBI database, revealing high similarity (100%) to sequences with the following accession numbers: CP026662.1, NR112116.1, CP002468.1, CP011686, MT605298.1, and CP011051, respectively. Five identified *B. subtilis* strains were used for further studies.

### Selection of B. subtilis strains based on probiotic properties

The ability of probiotic strains to produce extracellular enzymes is a crucial probiotic trait that aids digestion and reduces fecal waste [23]. All selected *B. subtilis* strains exhibited the capacity to produce key digestive enzymes, including amylase (starch hydrolysis), protease (protein hydrolysis), and lipase (lipid hydrolysis).

**Table 2.** Identification of *B. subtilis* strains by 16S rRNA gene sequencing.

Isolate	*Bacillus subtilis* strain	Similarity ratio (%)	GenBank ID of reference strain
BA07	*Bacillus subtilis *strain H1	100	CP026662.1
BA16	*Bacillus subtilis *strain IAM 12118	100	NR112116
BA29	*Bacillus subtilis *strain BSn5	100	CP002468.1
BA38	*Bacillus amyloliquefaciens *strain G341	100	CP011686
BA79	*Bacillus subtilis subsp. *strain JM9	100	MT605298.1
BA81	*Bacillus subtilis *strain T30	100	CP011051

**Table 3.** Probiotic characteristics of selected *B. subtilis* strains.

Properties	BA07	BA29	BA79	BA16	BA81
Extracellular enzyme production (U/ml)					
amylase	0.43^a^ ± 0.02	0.36^ab^ ± 0.032	0.34^ab^ ± 0.02	0.34^ab^ ± 0.02	0.30^b^ ± 0.02
protease	1.78^c^ ± 0.25	0.93^d^ ± 0.04	3.62^b^ ± 0.27	5.80^a^ ± 0.31	0.73^d^ ± 0.04
lipase	0.08^a^ ± 0.01	0.02^c^ ± 0.03	0.02^c^ ± 0.02	0.08^a^ ± 0.004	0.04^bc^ ± 0.02
Hydrophobicity (%)	55.31^b^ ± 2.08	60.24^ab^ ± 7.56	66.98^a^ ± 4.88	54.83^b^ ± 1.55	47.17^c^ ± 1.41
Co-aggregation (%)					
with *S. typhimurium*	38.87^a^ ± 5.15	43.98^a^ ± 6.44	39.71^a^ ± 5.15	41.45^a^ ± 4.01	29.86^b^ ± 4.18
with *E. coli* FG31-1	42.13^a^ ± 4.01	31.55^b^ ± 6.19	34.52^b^ ± 6.26	28.97^b^ ± 4.42	30.36^b^ ± 4.01
Self-aggregation (%)	62.31^a^ ± 2.22	56.74^a^ ± 3.34	44.72^b^ ± 2.37	35.58^b^ ± 4.5	23.21^c^ ± 3.74
Acid tolerance (%)					
at pH = 2	83.91 ± 3.61	85.3 ± 2.92	83.33 ± 2.53	84.48 ± 2.11	84.78 ± 2.24
at pH = 3	89.06 ± 2.24	90.95 ± 3.33	87.22 ± 2.84	90.17 ± 2.01	87.78 ± 3.28
Bile salt tolerance (%)					
at 0.3%	96 ± 1.8	95.5 ± 1.4	96.8 ± 0.9	94.3 ± 1.2	85.1 ± 1.0
at 0.5%	82.4 ± 2.1	82.7 ± 1.6	82.6 ± 1.4	83.5 ± 2.1	78.2 ± 2.3
Total high probiotic properties*	5	5	3	4	1

*Note:* Values with different superscripts (a-d) indicate statistically significant differences (*p* < 0.05); *values with superscripts are a or b.

Strain BA07 displayed the highest amylase activity (0.43 U/ml), which was significantly higher (*p* = 0.016) than that of strain BA81 (0.30 U/ml). Strain BA16 exhibited superior protease activity (5.80 U/ml, *p* < 0.05) compared to all other strains, while strains BA29 and BA81 showed the lowest protease activity (0.73–0.93 U/ml). Lipase activity was significantly higher (*p* < 0.05) in strains BA07 and BA16 (0.08 U/ml) compared to the other strains (0.02–0.04 U/ml).

The production of extracellular enzymes, such as amylase and protease, is a key characteristic of *B. subtilis* strains that contribute to their widespread industrial applications [24]. The ability of *B. subtilis* to secrete these enzymes is attributed to its sophisticated protein secretion systems (primarily Sec and Tat) and transcriptional regulatory mechanisms (including sigma factors and two-component systems) [25]. These systems coordinately regulate the production and release of enzymes, enabling *B. subtilis* to adapt to its environment and compete with other microorganisms.

The presence of starch, protein, and fat in poultry feed necessitates the production of amylase, protease, and lipase for efficient digestion. Furthermore, efficient fat digestion is crucial for mitigating the incidence of diarrhea [26]. Therefore, probiotics intended for use in poultry to prevent gastrointestinal diseases should ideally possess the ability to produce all three enzymes.

Cell surface hydrophobicity influences the overall adhesion capacity of bacteria and can facilitate interaction between probiotic bacteria and the host’s epithelial cells. This allows probiotic bacteria to compete with pathogens and produce digestive enzymes and indicates a greater ability of bacteria to adhere to the intestinal mucosa [27]. In this study, strain BA79 exhibited the highest hydrophobicity (66.98%), which was significantly higher (*p *< 0.05) than that of the other strains. Our results are consistent with those of Shahbaz et al. [28], who reported that *B. subtilis* strains isolated from chicken intestines exhibited hydrophobicity with water contact angles ranging from 62% to 68%.

The ability of probiotic bacteria to form cellular aggregates through auto-aggregation or co-aggregation enhances their persistence in the gut and can also antagonize pathogenic microorganisms [29]. Strain BA07 exhibited superior co-aggregation ability with both pathogens (38.87%–42.13%) compared to the other strains. Strain BA81 showed the lowest co-aggregation ability (29.86%–30.36%). Strain BA29 displayed the highest co-aggregation ability with *S. typhimurium* (43.98%) but low co-aggregation with *E. coli* FG31-1 (31.55%).

Similar to our findings, Ogbuewu et al. [30] reported that *B. subtilis* and *B. amyloliquefaciens* isolated from chickens exhibited auto-aggregation abilities of approximately 77% and 72%, respectively, after 2 h. These strains also demonstrated good co-aggregation abilities with other bacteria, reaching approximately 70%. Auto-aggregation in *B. subtilis* is primarily regulated by cell surface hydrophobicity and surface proteins [20]. Co-aggregation in *B. subtilis* spp. with other bacteria is modulated by environmental factors and interactions between surface proteins and polysaccharides.

The ability to withstand acidic conditions and bile salts is essential for probiotic bacteria to survive in the challenging environment of the chicken gastrointestinal tract, making tolerance to low pH and bile salts a fundamental requirement when selecting probiotic strains for animal feed [31]. In this study, the tolerance of *B. subtilis* strains to simulated gastric juice (pH 2 and pH 3) and bile salt conditions (0.3% and 0.5%) mimicking those encountered in the chicken small intestine was evaluated. As proposed by Prabhurajeshwar and Chandrakanth [32], bacteria of host origin often exhibit superior adaptation to the digestive conditions of their host, facilitating more effective colonization compared to bacteria from other sources. Consistent with this notion, all *B. subtilis* strains in this study demonstrated robust tolerance to both acidic and bile salt conditions, exhibiting high survival rates after 3 h of exposure to acidic environments (83.33%–90.95%) and 4 h of exposure to bile salt environments (82.66%–96.75%). These findings align with those of Penaloza-Vazquez et al. [33], who reported that *B. subtilis* strains exhibit good tolerance to pH 2.5–3.0 and can survive in the acidic conditions of the chicken stomach. The acid tolerance of *B. subtilis* can be attributed to its sophisticated intracellular pH regulatory systems, which include proton pumps, ion exchange mechanisms, and the production of intracellular buffers, enabling it to maintain a stable intracellular pH in acidic environments [34]. Furthermore, some *B. subtilis* strains possess the ability to produce bile salt hydrolase, an enzyme that hydrolyzes bile salts, mitigating their toxicity to bacterial cells [31].

Based on a comprehensive evaluation of probiotic properties, strains BA7 (*B. subtilis* strain H1) and BA29 (*B. subtilis* strain BSn5) displayed superior characteristics compared to the other strains (5 *vs.* 1–4 properties; Table 3) and were therefore selected for further evaluation in the production of experimental biopreparations.

### Viability of probiotic bacteria during storage

Freeze-drying is a widely employed method for preserving probiotic bacteria; however, this process can induce stress that can compromise bacterial viability. To mitigate this, various studies have utilized cryoprotective agents such as skim milk, whey protein, sugars, and biopolymers to enhance the survival rate of probiotics during freeze-drying [35].

Maintaining a sufficient number of viable cells in probiotic preparations is crucial for ensuring their efficacy in animal feed applications. A common starting point of probiotics is approximately 109 CFU/gm; however, this number can significantly decrease to 103–106 CFU/gm during storage [36].

**Figure 4.**
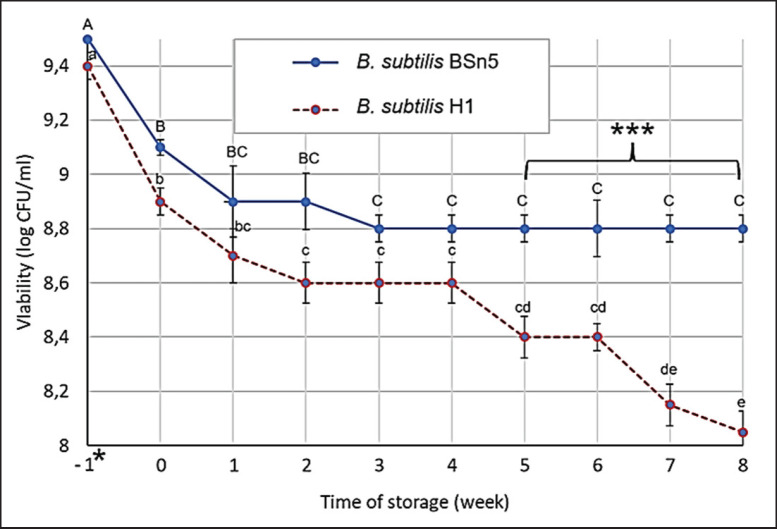
Viability of probiotic strains during storage. *Indicates the number of viable bacteria before and after freeze-drying; ***indicates statistically significant differences (*p* < 0.05) between *B. subtilis* strains within the same storage week; values with different letters (a–e, A–C) indicate statistically significant differences (*p* < 0.05) within the same *B. subtilis* strain.

Figure 4 illustrates the viability of the two selected probiotic strains in the fermented preparation after 2 months of storage at 4°C. The initial concentrations of both *B. subtilis* BSn5 and *B. subtilis* H1 were approximately 9.4–9.5 log_10_ CFU/ml. Following freeze-drying, the concentration decreased slightly to 9.2 log_10_ CFU/ml for *B. subtilis* BSn5 and 8.8 log_10_ CFU/ml for *B. subtilis* H1. During the first week of storage, a significant decrease in the concentration of both strains was observed (*p *< 0.05). However, from the second week onward, the concentration of *B. subtilis* BSn5 remained stable at around 8.8 log_10_ CFU/ml, while *B. subtilis* H1 exhibited a gradual decline, reaching approximately 8.0 log_10_ CFU/ml at week 8. The difference in viability between the two strains became statistically significant (*p *< 0.05) from week 5 onward.

These results indicate that *B. subtilis* BSn5 exhibits superior viability compared to *B. subtilis* H1 during storage, particularly over the long term, suggesting that *B. subtilis* BSn5 is a more promising candidate for the production of biopreparations.

### Viability of bacteria in the chicken gut

Throughout the 72 h (3-day) monitoring period following oral administration of *B. subtilis* at a dose of 109 CFU/ml, the chickens exhibited no abnormal clinical signs and maintained normal feed intake. This observation indicates that the *B. subtilis* strains used in this study are safe for chickens.

**Figure 5.**
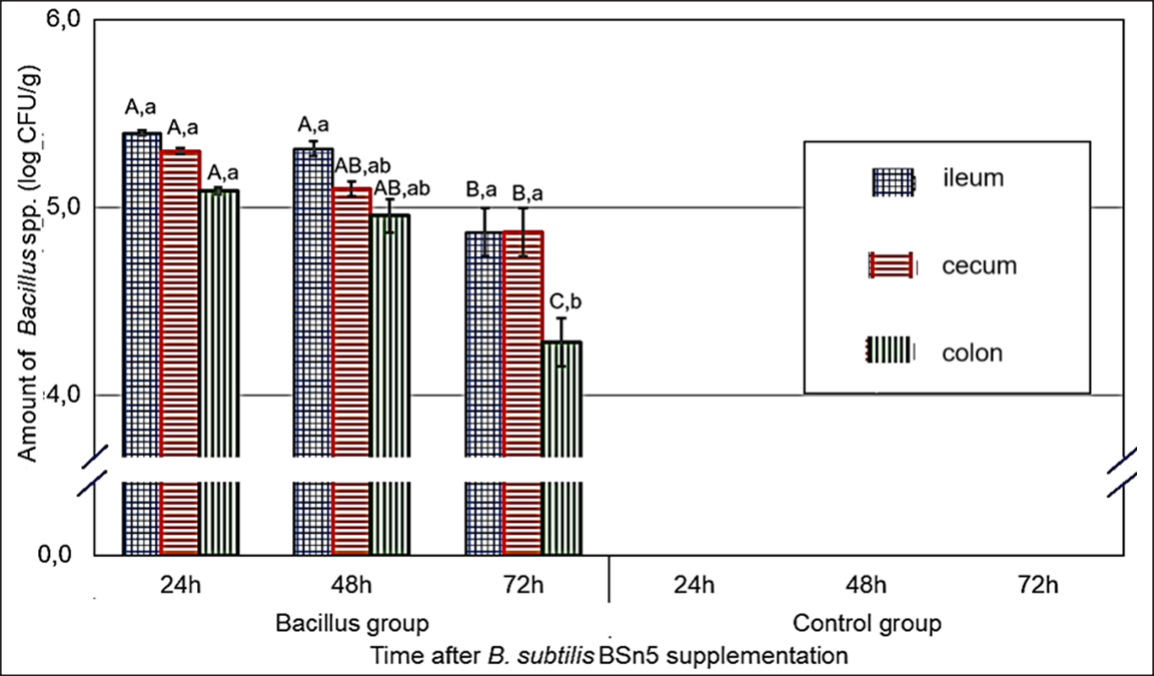
Persistence of *B. subtilis* BSn5 in the ileum, cecum, and colon of chickens at different time points after probiotic administration.

Figure 5 depicts the persistence of *B. subtilis* BSn5 in the ileum, cecum, and colon of chickens at 24, 48, and 72 h post-administration. Overall, the viability of *B. subtilis* BSn5 in the chicken gut decreased gradually over time. While the strain exhibited relatively good persistence in the first 24 h (4.09–4.20 log_10_ CFU/gm), a significant decline was observed at 48 h (3.36–3.60 log_10_ CFU/gm) and 72 h (2.88–3.11 log_10_ CFU/gm). Furthermore, the concentration of *B. subtilis* BSn5 was higher in the ileum and cecum compared to the colon (4.87–5.40 log_10_ CFU/gm *vs*. 4.28–5.09 log_10_ CFU/gm). This observation can be attributed to the rapid transit of digesta through the ileum, coupled with the presence of natural defense mechanisms such as digestive enzymes and bile, which may contribute to the faster decline in bacterial viability in this segment. In contrast, the cecum provides a more stable environment and harbors a diverse indigenous microbiota that may offer support and facilitate the persistence of *B. subtilis*.

As noted by Chandrasekaran et al. [37], probiotics are often transient inhabitants of the gut, persisting only for a limited time, and are not considered permanent members of the host’s microbiota. Therefore, regular probiotic supplementation at appropriate concentrations is recommended to maintain their beneficial effects.

## Conclusion

This study successfully isolated and characterized *B. subtilis* BSn5 from indigenous chicken feces, demonstrating its potential as an antibiotic alternative in poultry production. This strain not only exhibited potent antibacterial activity against *E. coli* and *Salmonella* spp. but also possessed key probiotic properties, including the ability to produce extracellular enzymes, cell surface hydrophobicity, auto-aggregation and co-aggregation capabilities, and tolerance to the harsh conditions of the gastrointestinal tract. Furthermore, this probiotic strain demonstrated good viability during storage. *In vivo* studies revealed that while *B. subtilis* BSn5 can survive in the chicken gut, its persistence is transient, necessitating regular supplementation to maintain its beneficial effects. Further *in vivo* studies in chickens are warranted to confirm its efficacy and develop more stable biopreparations.

## List of abbreviations

Bp, base pair; BLAST, basic local alignment search tool; CFU, colony forming unit; DIZ, diameter of inhibition zone; DNA, deoxyribonucleic acid; LB, lysogeny broth; MRS, de man–rogosa–sharpe; NCBI, national center for biotechnology information; PCR, polymerase chain reaction; rRNA, ribosomal ribonucleic acid; WHO, world health organization.
